# Permissive hypotension compared to fluid therapy for the management of traumatic haemorrhage: a rapid review

**DOI:** 10.29045/14784726.2022.12.7.3.34

**Published:** 2022-12-01

**Authors:** Rebecca Clarke, Enrico Dippenaar

**Affiliations:** Anglia Ruskin University; Anglia Ruskin University ORCID iD: https://orcid.org/0000-0001-8406-7373

**Keywords:** fluid therapy, permissive hypotension, traumatic haemorrhage

## Abstract

**Background:**

Haemorrhage and subsequent hypovolemia from traumatic injury is a potentially reversible cause of cardiac arrest, as interventions can be made to increase circulatory volume and organ perfusion. Traditionally, intravenous (IV) fluid therapy is recommended for all patients who have experienced a haemorrhagic emergency. There has been some argument, however, that this may not be the most effective treatment as isotonic fluids can dilute coagulation factors and further stimulate bleeding. Permissive hypotension, also known as hypotensive resuscitation within the context of damage control resuscitation, is a method of managing haemorrhagic trauma patients by restricting IV fluid administration to allow for a reduced blood pressure. It is important to evaluate and compare current research literature on the effects of both permissive hypotension and fluid therapy on patients suffering from traumatic haemorrhage.

**Methods:**

A rapid review was conducted by systematically searching and identifying literature to narratively compare permissive hypotension and fluid therapy. Searches were carried out across two databases to find relevant primary research containing quantitative data that provide contextual and statistical evidence to achieve the aim of this review. Papers were narratively synthesised to produce key themes for discussion.

**Results:**

The database searches identified 125 records, 78 from PubMed and 47 from ScienceDirect. Eleven duplicates were removed, and 114 titles screened. Ninety-four records were initially excluded and nine more after abstract review. Eleven papers were critiqued using Benton and Cormack's framework, with eight articles included in the final review.

**Conclusion:**

Permissive hypotension may have a positive impact on 30-day mortality, when compared with fluid resuscitation methods, however there is evidence to suggest that hypotensive resuscitation may be more effective for blunt force injuries. Some studies even suggest a reduction in the treatment cost when reducing fluid volumes. Penetrating injuries are usually more likely to be a compressible source of haemorrhage within which haemorrhage control can be gained much more easily. There are recommendations for the use of permissive hypotension in both compressible and non-compressible injuries. It is difficult at this time to draw definitive conclusions for the treatment of every case related to traumatic haemorrhage given the variability and unpredictability of trauma.

## Background

Traumatic injuries are responsible for 4.4 million (8%) deaths per year around the world, of which it constitutes three of the top five causes for people between 5 and 29 years old (World Health Organization, 2021). Haemorrhage occurs when blood vessels are ruptured by laceration, blunt force or crushing injuries (Curry et al., 2011). Blood haemorrhages out of the damaged vessel, causing hypovolemia as the overall intravascular blood volume is reduced (Waugh & Grant, 2014). Haemorrhage can occur internally, where internal blood vessels are ruptured and blood pools within the body cavities, or externally when blood is lost outside of the body (Johnson & Burns, 2020). In severe cases, both forms of haemorrhage can lead to difficulties in maintaining homeostasis as the body is dependent on the oxygen-carrying capacity of haemoglobin within the blood (Waugh & Grant, 2014). The most important factor in managing critical levels of blood loss is the prevention of further bleeding, as critical levels of blood loss result in the body being unable to maintain organ perfusion and subsequently lead to cardiac arrest (Taghavi & Askari, 2020). Hypovolemia is a potentially reversible cause of cardiac arrest, as interventions can be made to increase circulatory volume, which will improve cardiac output and organ perfusion (Soar et al., 2021).

Traditionally, intravenous (IV) fluid therapy is recommended for all patients who have experienced a haemorrhagic emergency and who present with hypotension, with the aim of increasing circulatory volume (Joint Royal Colleges Ambulance Liaison Committee & Association of Ambulance Chief Executives, 2020). There has been some argument, however, that this may not be the most effective treatment in the pre-hospital setting as sodium chloride (the fluid in common use in the United Kingdom) can dilute coagulation factors and further stimulate bleeding (Fisher & Carius, 2018). Permissive hypotension, known as hypotensive resuscitation within the context of damage control resuscitation, is a method of managing haemorrhagic trauma patients by restricting IV fluid administration to allow for a reduced blood pressure (Kudo et al., 2017). Hypotension is generally considered to be a systolic blood pressure (SBP) below 90 mmHg with a diastolic blood pressure below 60 mmHg, or a mean arterial pressure (MAP) below 70 mmHg (Sharma et al., 2021). Permissive hypotension aspires to balance organ perfusion and haemostasis (Kudo et al., 2017). Given that 40% of traumatic deaths are due to uncontrolled bleeding, it is important to evaluate and compare current research literature on the effects of both permissive hypotension and fluid therapy, and how it could be used to manage patients suffering from traumatic haemorrhage.

## Methods

A rapid review was conducted by systematically searching and identifying literature that could be used to fulfil the aim of this article, which is to review and narratively compare permissive hypotension with fluid therapy for the management of traumatic haemorrhage. Literature searches were carried out across two databases between 22 and 28 February 2021. PubMed and ScienceDirect were chosen for the literature searches due to their medical relevance and the variety of research available on the databases (Williamson & Minter, 2019). Both databases were available with institutional access through Anglia Ruskin University’s library website (Anglia Ruskin University, 2021).

Several key words were used based on the PICO (patient, intervention, comparison, outcomes) framework and combined using Boolean operators and search methods such as phrasing and truncation. Synonyms of the key words were also then considered and smaller combination word strings were used between the headings to search for literature in the databases. Table 1 describes the four headings as they relate to the PICO groups and the words used within each.

**Table 1.** Keywords used in the literature search.

Patient group	Non-IV fluid	IV fluid	Outcome
Trauma* Injur* Haemorrhage Hemorrhage Bleeding Hypovolem*	‘Permissive Hypotension’ ‘Hypotensive Resuscitation’	Fluid ‘Fluid Therapy’	Mortality Survival Outcome

Words were combined using OR within columns, AND between columns. ‘Phrase searching’ and *Truncation was also used. IV: intravenous.

The databases were searched to find relevant primary research containing quantitative data that provide contextual and statistical evidence to achieve the aim of this review (Griffiths, 2011). Randomised control trials and clinical trials were chosen for the review as these are considered to have high reliability and validity within the hierarchy of evidence (Creswell & Creswell, 2018). Table 2 describes the inclusion and exclusion criteria used during the screening process. All papers considered during full-text screening were further evaluated against the Benton and Cormack critiquing framework to identify the levels of rigour between the studies (Benton & Cormack, 1996). Papers with low rigour were excluded from the study. The final papers were narratively synthesised to produce key themes for discussion.

**Table 2.** Inclusion and exclusion criteria.

**Inclusion**
Written in English – for clarity and understanding. Full text available – to consider the entire research paper. Animal studies – to allow for a holistic review of treatment and experimentation.
**Exclusion**
Paediatrics – they have differences in their anatomy and physiology which would introduce further confounding variables. Traumatic brain injuries – these types of traumatic injury have presentations that are often very different to traumatic haemorrhage, e.g. patients with traumatic brain injuries may be hypertensive. Studies also researching drug administration – these would not be able to provide a direct comparison between permissive hypotension and fluid resuscitation. Low rigour – papers with low rigour as determined through critique screening would not provide valuable evidence for comparison. Reviews – only primary research papers were used to determine the source material of the data.

Limitations of this review include the potential for studies to have been missed when carrying out the search as only two databases were used. Similarly, the studies were narratively synthesised by a single researcher (RC); therefore the discussion points may be influenced by researcher bias. This means the results of this review may be difficult to generalise. However, there is confidence that the elements described in this review provide a good rapid assessment of the literature within this field and the current state of research and clinical practice.

## Results

**Figure 1:**
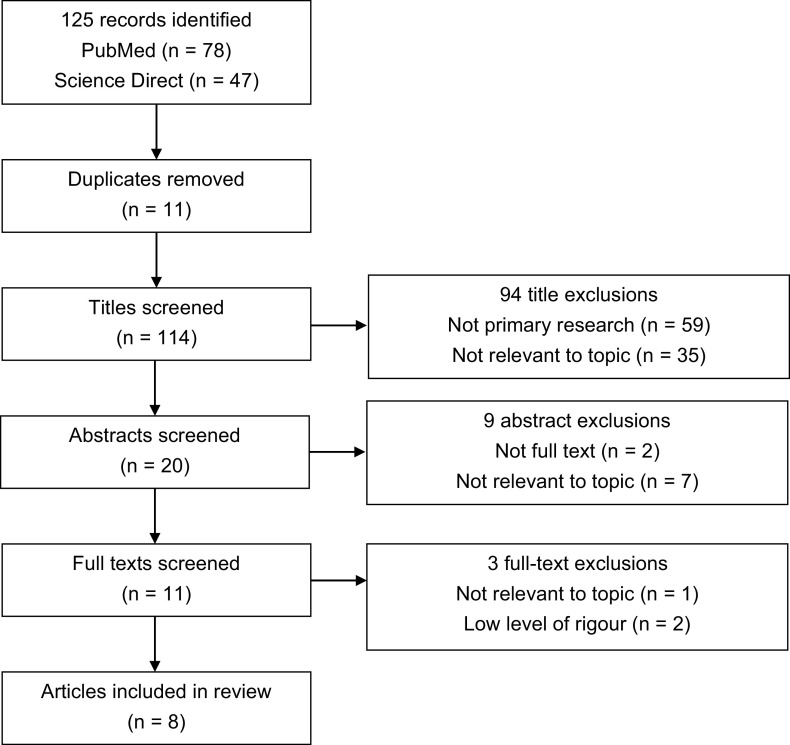
Figure 1. PRISMA flow diagram of the screening process.

The database searches identified 125 records, 78 from PubMed and 47 from ScienceDirect (Figure 1). Eleven duplicates were removed, and 114 titles screened against the inclusion criteria. Ninety-four records were excluded based on relevance to the topic and not being primary research studies. Abstract screening further excluded nine records, two of which were excluded due to a lack of full text and seven due to irrelevance to the topic. Eleven papers were full-text reviewed and critiqued, with one being excluded based on relevance and two excluded due to a low level of rigour. Eight papers were included in the final review (Table 3).

**Table 3.** Summary of selected studies included in the review.

Author, year, title	Methods	Results	Conclusion
Bickell et al. (1994), ‘Immediate versus delayed fluid resuscitation for hypotensive patients with penetrating torso injuries’	Study design: Randomised control trial. Location: One emergency medical service and one hospital in Houston, Texas, USA. Description: 598 patients with penetrating trauma to the torso and SBP < 90 mmHg were randomised. They were either in the group that received immediate fluid resuscitation with a standard paramedical protocol, that included rapid infusion of isotonic crystalloid, or in the delayed-resuscitation group who were cannulated but did not receive fluids until they were in the operating room. Once in the operating room all patients were treated as necessary to maintain systolic arterial pressure of 100 mmHg.	70% of delayed fluid resuscitation patients survived to discharge compared to 62% of immediate fluid resuscitation patients. Estimated blood loss was similar in the two groups. 23% patients in the experimental group had one or more complications compared with 30% in the immediate-resuscitation group. The duration of hospitalisation was shorter in the delayed-resuscitation group.	For hypotensive patients with penetrating torso injuries, delay of aggressive fluid resuscitation is recommended until operative intervention improves the outcome.
Capone et al. (1995), ‘Uncontrolled hemorrhagic shock outcome model in rats’	Study design: Experimental study. Location: University of Pittsburgh Medical Centre, Pittsburgh, USA. Description: 40 rats were used to simulate the life-support chain of emergency medical services for trauma by inducing UHS. The rats were randomised into four groups: group 1 received no FR; group 2 received no FR for 60 min, then all-out hospital FR; group 3 received LR IV starting at 15 min after tail amputation, titrated to maintain MAP at 40 mmHg until 60 min and then all-out hospital FR; group 4 received the same as group 3, but sustaining a MAP at 80 mmHg, the normal value for rats.	Survival to 72 hours was achieved in none of the rats in group 1; 9 of 10 in group 2; all 10 in group 3; and 7 of 10 in group 4 (p < 0.05). Of the 26 survivors to 72 hours, 24 showed ND scores between 0 and 10%, with no difference between groups.	This model development confirms not only the problem with FR aiming at normotension before control of bleeding, but also the value of FR after control of bleeding. Considering the results of this report, the next logical step will be to study pre-hospital FR to MAP 40 vs 80 mmHg, using only LR.
Durusu et al. (2010), ‘Comparison of permissive hypotensive resuscitation, low-volume fluid resuscitation, and aggressive fluid resuscitation therapy approaches in an experimental uncontrolled haemorrhagic shock model’	Study design: Experimental study. Location: Kirikkale University Faculty of Medicine, Kirikkale, Turkey. Description: 44 guinea pigs were split into six groups including normovolemic-normotensive fluid treatment group, normovolemic-permissive hypotensive fluid treatment group, low-volume normotensive fluid treatment group, low-volume permissive hypotensive fluid treatment group, no treatment and sham-operated groups. Resuscitation was initiated when MAP reached 30 mmHg. In the permissive hypotensive resuscitation group, fluid treatment continued until MAP reached 45+/-5 mmHg and in the aggressive fluid groups until MAP reached 60+/-5 mmHg.	Mean survival time was 122.75 mins in the normovolemic-normotensive fluid group 130.87 mins in the normovolemic-permissive hypotensive group, 122.12 mins in the low-volume-normotensive fluid group, and 152.25 mins in the low-volume-permissive hypotensive fluid group. Survival time was significantly higher in the group in which low-volume-permissive hypotensive fluid treatment was applied than in the other groups (p = 0.036). When pressure effect was compared during treatment, permissive-hypotensive resuscitation was more effective in both groups that received colloid and crystalloid treatment.	When pressure effect was compared during treatment, permissive-hypotensive resuscitation was more effective in both groups that received colloid and crystalloid treatment.
Dutton et al. (2002), ‘Hypotensive resuscitation during active haemorrhage: impact on in-hospital mortality’	Study design: Randomised control trial. Location: University of Maryland School of Medicine, Baltimore, Maryland, USA. Description: 110 patients presenting in haemorrhagic shock with SBP < 90 mmHg were randomised to one of two FR protocols: target SBP > 100 mmHg (conventional) or target SBP 70 mmHg (low). Fluid therapy was titrated to this endpoint until definitive haemostasis was achieved. In-hospital mortality, injury severity and probability of survival were determined for each patient.	There was a significant difference in SBP observed during the study period (114 mmHg vs 100 mmHg, p < 0.001). ISS and the duration of active haemorrhage were not different between groups. Overall survival was 92.7%, with four deaths in each group.	Titration of initial fluid therapy to a reduced SBP during active haemorrhage did not affect mortality in this study. Reasons for the decreased overall mortality and the lack of differentiation between groups likely include the heterogeneous nature of human traumatic injuries, and the imprecision of SBP as a marker for tissue oxygen delivery.
Geeraedts et al. (2015), ‘Prehospital fluid resuscitation in hypotensive trauma patients: do we need a tailored approach?’	Study design: Retrospective analysis. Location: Liverpool Hospital, New South Wales, Australia. Description: Retrospective analysis of 941 trauma patients from the Southwestern Sydney Regional Trauma Registry with field hypotension presenting to a level 1 trauma centre. Regression models were used to investigate associations between pre-hospital fluid volumes, shock index and blood transfusion in the ED and mortality at 24 hours.	A 1 l increase of pre-hospital IV fluid was associated with a 7% decrease of shock index in the ED (p < 0.001). Volumes of 0.5–1.0 l and 1.0–2.0 l were associated with reduced likelihood of shock (OR 6.1, p = 0.03). Volumes of 1.0–2.0 l were also associated with an increased likelihood of receiving blood transfusion in ED (OR 3.27, p < 0.001). Patients who had received volumes of > 2.0 l have a much greater likelihood of receiving blood transfusion in ED (OR 9.92, p < 0.001). Mortality at 24 hours was not associated with pre-hospital IV fluids.	Decision-making regarding pre-hospital IV FR is critical and may need to be tailored to the individual situation. Further research is needed to clarify whether a causal relationship exists between pre-hospital IV fluid volume and blood transfusion.
Haut et al. (2011), ‘Prehospital intravenous fluid administration is associated with higher mortality in trauma patients: a National Trauma Data Bank analysis’	Study design: Retrospective cohort study. Location: 600 US trauma centres. Description: A five-year retrospective analysis of 776,734 patients from the National Trauma Data Bank. Multiple logistic regression was used with mortality as the primary outcome measure. Patients with pre-hospital IV fluid administration were compared to those without pre-hospital IV fluid administration, using patient demographics, mechanism, physiologic and anatomic injury severity and other pre-hospital procedures as co-variates. Subset analysis was performed based on mechanism (blunt/penetrating), hypotension, immediate surgery, severe head injury and ISS.	Mortality was significantly higher in patients receiving pre-hospital IV fluids (4.8% vs 4.5%, p = 0.0001). Multi-variable analysis demonstrated that patients receiving IV fluids were significantly more likely to die (OR 1.11, 95% CI). The association was identified in nearly all subsets of trauma patients. It is especially marked in patients with penetrating mechanism (OR 1.25, 95% CI), hypotension (OR 1.44, 95% CI), severe head injury (OR 1.34, 95% CI) and patients undergoing immediate surgery (OR 1.35, 95% CI).	The harm associated with pre-hospital IV fluid administration is significant for victims of trauma. The routine use of pre-hospital IV fluid administration for all trauma patients should be discouraged.
Morrison et al. (2011), ‘Hypotensive resuscitation strategy reduces transfusion requirements and severe postoperative coagulopathy in trauma patients with haemorrhagic shock: preliminary results of a randomised control trial’	Study type: Randomised control trial. Location: Ben Taub General Hospital, Houston, Texas, USA. Description: 90 patients aged between 11 and 45 years in haemorrhagic shock who required emergency surgery were randomised to one of the two arms of the study for intraoperative resuscitation; LMAP of 50 mmHg or HMAP of 65 mmHg.	The LMAP group received significantly fewer blood products and total IV fluids during intraoperative resuscitation (2898 ml vs 1594 ml, p = 0.03) and had significantly lower mortality than those in the HMAP group (p = 0.03).	Hypotensive resuscitation is a safe strategy for use in the trauma population. Specifically, resuscitating patients with the intent of maintaining a target minimum MAP of 50 mmHG, rather than 65 mmHG, significantly decreases post-operative coagulopathy and early death.
Schreiber et al. (2015), ‘A controlled resuscitation strategy is feasible and safe in hypotensive trauma patients: results of a prospective randomised pilot trial’	Study type: Randomised control trial. Location: 19 EMS and 10 regional hospitals across the United States and Canada. Description: 192 blunt and penetrating trauma victims with SBP < 90 mmHg were randomised into the CR and SR groups. CR patients received 250 ml fluid boluses to maintain SBP of 70 mmHg. The SR group patients received 2.0 l bolus and additional fluid as needed to maintain a SBP of 110 mmHg.	The mean crystalloid volume administered was 1.0 l in the CR group and 2.0 l in the SR group. At 24 hours after admission, there were 5% deaths in the CR group and 15% in the SR group. Among patients with blunt trauma, 24-hour mortality was 3% (CR) and 18% (SR). There was no difference in mortality among patients with penetrating trauma.	CR is achievable in out-of-hospital and hospital settings and may offer an early survival advantage in blunt trauma. A large-scale, Phase III trial to examine its effects on survival and other clinical outcomes is warranted.

CI: confidence interval; CR: controlled resuscitation; ED: emergency department; EMS: emergency medical services; FR: fluid resuscitation; HMAP: higher mean arterial pressure; IV: intravenous; ISS: injury severity score; LMAP: lower target mean arterial pressure; LR: lactated Ringer’s solution; MAP: mean arterial pressure; ND: neurological deficit; OR: odds ratio; SBP: systolic blood pressure; SR: standard resuscitation; UHS: uncontrolled haemorrhagic shock.

## Discussion

Following a narrative synthesis of the articles included in this review, four key themes were identified and discussed further to understand how permissive hypotension compares to fluid therapy in the management of traumatic haemorrhage. The key themes identified are mortality and morbidity, volume of fluids and cost, compressible versus non-compressible haemorrhage and the unpredictability of trauma.

### Mortality and morbidity

The most important thing to consider when comparing two treatment methods is the long-term effects they have on the patient (Griffiths, 2011). Mortality and morbidity are the key indicators in assessing the effectiveness of a treatment (Griffiths, 2011). Morrison et al. (2011) conducted a randomised control trial comparing hypotensive and normotensive resuscitation and specifically focused the findings on mortality and post-operative complication over a 30-day period following the treatment. As a result, Morrison et al. (2011) found that patients who had a target MAP of 50 mmHg had significantly lower mortality rates after 30 days compared with those who had a target MAP of 65 mmHg. The hypotensive group also received fewer blood and fluid products and were less likely to develop post-operative complications. Therefore, it was concluded that permissive hypotension is safe and effective in trauma care. On the other hand, Dutton et al. (2002) also carried out a randomised control trial, looking at the impact of hypotensive resuscitation during active haemorrhage on mortality, and found that overall survival did not significantly differ between the normotensive and hypotensive groups, with a 92.7% survival rate for each. Both studies were conducted over a similar time period, and with a similar number of participants, however Dutton et al. (2002) chose to focus only on penetrating trauma while Morrison et al. (2011) studied patients who had experienced both blunt and penetrating trauma. This could suggest that hypotensive resuscitation may be more effective for blunt force injuries. However, Bickell et al. (1994) focused their study on patients with penetrating torso injuries and found there was a significant difference in mortality rates for those who received fluid immediately (62% survival rate) compared with those who received delayed fluid resuscitation (70% survival rate). It could therefore be suggested that fluids are important in penetrating injuries; however, they should be used cautiously to promote natural clotting and haemostasis.

### Volume of fluids and cost

When comparing permissive hypotension with fluid therapy, it is important to consider the wider implications of the treatment methods. Quite obviously the biggest difference between the two treatment methods is the administration of fluids and this can mean that normotensive fluid therapy can be costly (Varney & Guest, 2003). Geeraedts et al. (2015) used a retrospective analysis of trauma patients to understand the relationship between the volume of fluids infused in the pre-hospital environment and subsequent blood transfusions received in the emergency department (ED), as well as mortality after 24 hours. They found that patients who received less than one litre of fluid prior to arriving at the ED were far less likely to require a blood transfusion (odds ratio (OR) 3.27 p < 0.001) and those who received more than two litres of fluid were significantly more likely to require blood products (OR 9.92 p < 0.001). Based on the average cost of a 500 ml bag of 0.9% sodium chloride, and the cost of a blood transfusion, the fluid therapy treatment can cost upwards of £1000 per patient without considering the additional hidden costs of staff time and prolonged care (Farla Medical Ltd., 2021; Varney & Guest, 2003). Comparatively, when considering permissive hypotension in this study there was no difference in mortality at 24 hours and therefore it is arguable that this is an unnecessary cost. There are however some limitations to Geeraedts et al.’s (2015) study, for example as this was a retrospective analysis it is not possible to determine whether the fluid administration was the direct cause of the need for a blood transfusion, merely that there was an association between the two. This association could have been due to the injuries the patients received rather than the fluids, for example if the patient suffered more severe injuries, then they may have been given more fluids and needed the blood transfusion due to the injuries sustained. Schreiber et al. (2015) also monitored the volume of fluids patients received in their study. They found that when trying to maintain an SBP of over 100 mmHg the patients required an additional one litre of fluid compared with patients who were in the permissive hypotension group with a blood pressure sustained over 70 mmHg. Patients in the permissive hypotensive group who had experienced blunt force trauma had better outcomes than those in the standard resuscitation group, and there was no difference in the outcomes for patients with penetrating trauma. This reinforces the argument that this fluid is an unnecessary cost.

### Compressible versus non-compressible haemorrhage

Several of the papers identified that permissive hypotension was beneficial prior to haemorrhage control, and fluid resuscitation was beneficial after this was achieved. It is therefore important to consider traumatic haemorrhage that is compressible, versus haemorrhage which is not. Compressing the source of bleeding is the first-line treatment for haemorrhage control, and therefore compressible injuries can be managed much more effectively, for example haemorrhage from a limb (Donley & Loyd, 2021). Non-compressible haemorrhage, such as bleeding into the pelvis, is much harder to manage and therefore we must consider the benefits and risks of permissive hypotension in these injuries (Donley & Loyd, 2021). Capone et al. (1995) focused their study on compressible haemorrhage by amputating rats’ tails. Their study primarily concentrated on the effects of permissive hypotension on uncontrolled haemorrhagic shock, however they also discussed the value of fluid resuscitation following haemorrhage control (Capone et al., 1995). Comparatively, Durusu et al. (2010) studied the effects of permissive hypotension on uncontrolled haemorrhagic shock in non-compressible injuries by inflicting internal abdominal lacerations on guinea pigs. This led them to conclude that permissive hypotension was beneficial in these types of injury as large fluid volumes were responsible for the prolonged time to achieve haemostasis (Capone et al., 1995). Haut et al. (2011) provided a holistic insight into the differences of hypotensive resuscitation in compressible and non-compressible injuries by including both blunt (non-compressible) and penetrating (compressible) injuries in their review of the National Trauma Data Bank. They found that the administration of IV fluids led to an increased mortality rate in both types of injury, however patients suffering from penetrating injury were also more likely to be given fluids in the first instance (Haut et al., 2011).

### The unpredictability of trauma

The biggest limitation of any study or review related to traumatic injuries is the unpredictability of trauma. Due to the nature of traumatic injuries, it can be particularly difficult to study a single type of injury, in this case haemorrhage, as patients who have undergone trauma often have multiple injuries (Frink et al., 2017). This means that the treatment they require is considered on an individual basis and can reduce the population of eligible participants for trauma research (Frink et al., 2017). Similarly, it can be difficult to identify whether the independent variable is directly impacting the patient outcomes, or whether confounding variables are affecting the results. Capone et al. (1995) conducted one of the first studies surrounding permissive hypotension for uncontrolled haemorrhagic shock, with the aim of reducing some of the unpredictability by studying rats in a laboratory setting. All rats received the same tail amputation, at three quarters of its total length. However, Capone et al. (1995) found that in this preliminary study the tail amputation alone did not always produce a consistent and severe enough uncontrolled haemorrhage necessary for directly comparing the normotensive and hypotensive treatments. This demonstrates how despite all rats receiving the same injury, the unpredictability of trauma creates complications when testing treatments. A more refined final study was therefore carried out, in which the rats first underwent a controlled haemorrhage of 3 ml/100 g for 15 minutes prior to the three-quarter tail amputation (Capone et al., 1995). This method does not accurately represent real trauma; however, it is still important to consider these findings as the researchers were able to eliminate some of the unpredictability of trauma and it demonstrates how difficult it can be to study traumatic injuries. The refined study created results that were less varied and consequently can be used to draw comparisons between permissive hypotension and fluid therapy. Durusu et al. (2010) also conducted a laboratory-based study, in which guinea pigs were used, however their study had greater applicability to real life as uncontrolled haemorrhagic shock was achieved through internal injuries which are much more common, and potentially life threatening, than limb amputation (Johnson & Burns, 2020). This study therefore can be used to compare permissive hypotension and fluid resuscitation while considering the unpredictability of trauma. The remaining six papers, on the other hand, were all able to conduct their studies on humans despite the unpredictability of trauma. Haut et al. (2011) considered this and therefore conducted a retrospective analysis of trauma patients to assess the correlation between pre-hospital fluid administration and higher mortality rate. This method provided a very large sample population and included all severities of trauma which means the results can be generalised to the wider population. The study is, however, limited to the data available on the database used (Haut et al., 2011). For example, the National Trauma Data Bank does not report variables such as transport times to hospital, therefore the effect of IV fluid administration on mortality cannot be determined with complete reliability (Haut et al., 2011).

## Conclusion

Permissive hypotension may have a positive impact on 30-day mortality for patients experiencing both blunt and penetrating trauma, when compared with fluid resuscitation methods, however there is evidence to suggest that hypotensive resuscitation may be more effective for blunt force injuries. This could be because penetrating injuries are usually more likely to be a compressible source of haemorrhage within which haemorrhage control can be gained much more easily. There are recommendations for the use of permissive hypotension in both compressible and non-compressible injuries, the benefits of which are seen even more so once haemorrhage control has been achieved. In addition to the effects on mortality and morbidity, reducing the volume of fluids given to a patient can also substantially reduce the cost of treatment, leading to wider treatment options available in lower resourced settings. It is difficult at this time to draw definitive conclusions for the treatment of every case related to traumatic haemorrhage given the variability and unpredictability of trauma.

## Recommendations

The biggest factor to consider when questioning the reliability of research previously carried out into permissive hypotension is the unpredictability of trauma and the influence of confounding variables on the results. Therefore, more research needs to be done to consolidate the evidence and negate the impact of these external influences. This can be done by conducting a large-scale randomised control trial that directly compares permissive hypotension and fluid therapy treatment methods for the management of traumatic haemorrhage. The results of this study should focus on the condition of the patient at the time of injury, immediately after the injury and for a 30-day period following the trauma. Mortality rates, blood loss, length of time taken to control the haemorrhage and neurological deficits of the patients should all be examined to determine both the immediate and the long-term impacts of the treatment. The feasibility of such a study must be considered, however, as this study would require large amounts of time and the ethics of the different treatment methods would need to be considered. A smaller pilot study could be carried out first to assess this.

Permissive hypotension forms part of the damage control resuscitation guidance first issued by the navy (Leibner et al., 2020). Following the results of the recommended study, should it be confirmed that permissive hypotension is a safer and more effective treatment for paramedics to use in the pre-hospital environment, concise new guidelines should be compiled to make it clear how haemorrhaging trauma patients should be managed. These guidelines should highlight the role of permissive hypotension in damage control resuscitation, and further research into the use of damage control resuscitation in the pre-hospital setting could also then be carried out. These guidelines should be publicised, and staff should be given training to demonstrate the effectiveness of permissive hypotension to promote a positive culture surrounding the treatment.

## Author contributions

RC completed this review as part of an undergraduate dissertation, with ED as the supervisor. Both reviewed and approved the final manuscript. RC acts as the guarantor for this article.

## Conflict of interest

None declared.

## Funding

None.
